# Metabolic flexibility across the spectrum of glycemic regulation in youth

**DOI:** 10.1172/jci.insight.146000

**Published:** 2021-02-22

**Authors:** Fida Bacha, Sara Klinepeter Bartz, Maurice Puyau, Anne Adolph, Susan Sharma

**Affiliations:** 1 United States Department of Agriculture Agricultural Research Service (USDA/ARS) Children’s Nutrition Research Center, Texas Children’s Hospital, Baylor College of Medicine, Houston, Texas, USA.; 2 Division of Pediatric Endocrinology and Diabetes, Texas Children’s Hospital, Baylor College of Medicine, Houston, Texas, USA.

**Keywords:** Metabolism, Diabetes, Insulin, Obesity

## Abstract

**BACKGROUND:**

Metabolic flexibility (MF) refers to the relative ability to utilize lipid and carbohydrate substrates and to transition between them. It is not clear whether MF is impaired in obese youth and what the determining factors are.

**METHODS:**

We investigated the determinants of MF (increased respiratory exchange ratio [ΔRER] under insulin-stimulated conditions) in pubertal youth (*n* = 104; 15.6 ± 1.8 years) with obesity across the spectrum of glucose tolerance compared with normal weight (NW) controls, including body composition (fat-free mass [FFM], %body fat), visceral adipose fat (VAT) (MRI), glycemia, and insulin sensitivity (IS) [3-hour hyperinsulinemic-euglycemic clamp with measurement of lipolysis ([^2^H_5_] glycerol), free fatty acids (FFAs), and RER (indirect calorimetry)].

**RESULTS:**

Youth with prediabetes and type 2 diabetes had lower ΔRER and oxidative and nonoxidative glucose disposal compared with NW, with no significant difference in ΔRER between NW and obese with normal glucose tolerance. In multiple regression analysis, IS_FFM_ (β = 0.4, *P* = 0.004), percentage suppression of FFAs (*r* = 0.26, *P* = 0.007), and race/ethnicity (β = –0.23, *P* = 0.02) contributed to the variance in ΔRER (*R*^2^ = 0.30, *P* < 0.001) independent of percentage body fat (or VAT), sex, Tanner stage, and hemoglobin A1c.

**Conclusion:**

MF is defective at the extreme of the metabolic phenotype in obese youth with dysglycemia related to a defect in IS limiting substrate utilization.

**FUNDING:**

USDA/ARS Project Number 3092-51000-057.

## Introduction

Energy metabolism involves uptake, distribution, and breakdown of nutrients to yield energy. The metabolic disturbances that occur with obesity are associated with a disturbance in utilization of metabolic fuels (1). Metabolic flexibility refers to the relative ability to utilize lipid and carbohydrate substrates and to transition between them (2). This is reflected in the switch from predominantly lipid oxidation during the postabsorptive fasting state to the suppression of lipid oxidation and increased glucose oxidation and storage under glucose and insulin-stimulated conditions (3, 4). Adults with type 2 diabetes have reduced metabolic flexibility compared with obese controls, attributed to a defect in glucose transport (5). At the cellular level, the underlying physiological disturbance has been related to impaired mitochondrial function (6–8), accumulation of toxic intracellular metabolites of fatty acyl-CoA, diacylglycerol, and ceramides, resulting in impaired insulin signaling (9). Insulin secretion is also dependent on the cell’s ability to select substrates for mitochondrial oxidative phosphorylation among glucose, lipids, and amino acids (10). This function may also be altered in the setting of “metabolic inflexibility” (11).

In the short term, healthy nondiabetic obese and lean adolescents adapt to changes in dietary carbohydrate and fat intake, appropriately adjusting their substrate oxidation rates to match the macronutrient intakes (12–14). It is unclear if this capacity deteriorates over time and if metabolic inflexibility is manifest in obese youth with dysglycemia compared with those with normal glucose tolerance (NGT). We previously reported lower insulin-stimulated oxidative and nonoxidative glucose disposal in obese youth with type 2 diabetes compared with nondiabetic controls of similar adiposity (15). We also demonstrated less suppression of fat oxidation in response to hyperinsulinemia in relationship to the degree of insulin resistance in youth with obesity (16). Recent studies demonstrate more profound insulin resistance in youth compared with adults of similar body adiposity and glycemic status (17, 18). Youth with type 2 diabetes also show more rapid deterioration of β cell function compared with adults with the disease (19). It is not clear if youth with type 2 diabetes or early dysglycemia have a defect in metabolic flexibility compared with insulin-resistant youth with obesity but with normoglycemia. The determinants of this defect in substrate utilization are also not clear. This is important to understand as it may influence therapeutic strategy for this defect that may eventually contribute to β cell dysfunction.

In this study, we investigated metabolic flexibility, defined as the increase in respiratory exchange ratio (ΔRER) and substrate utilization from baseline postabsorptive state to insulin-stimulated state in youth with normal weight (NW) and with obesity across the spectrum of glycemic regulation and determined the relationship between glucose and lipid metabolism and metabolic flexibility. We hypothesized that (a) youth with impaired glucose regulation (prediabetes and type 2 diabetes) have reduced metabolic flexibility compared with obese youth with normoglycemia and with normal weight peers and (b) this metabolic inflexibility in youth with impaired glucose regulation is determined by substrate availability in relation to reduced insulin sensitivity.

## Results

### Physical and metabolic characteristics of study participants.

The participants with NW and with obesity across the glycemic spectrum did not differ with respect to age, sex distribution, or Tanner stage. They were all pubertal, Tanner stages II–V (Table 1). There was a difference in race/ethnicity among the groups, with more Hispanic youth with type 2 diabetes. Therefore, we adjusted for race/ethnicity in subsequent analyses of metabolic variables. BMI *z* score, and measures of total body adiposity (%body fat, fat mass) or abdominal fat (waist circumference or visceral fat), were significantly higher in the youth with obesity compared with the NW group as expected, with no significant differences in these parameters among the NGT, prediabetes, or type 2 diabetes groups. HbA1c was significantly higher in the type 2 diabetes group compared with the NW and NGT groups. Adiponectin concentrations were significantly lower in the type 2 diabetes and prediabetes compared with NW and in the type 2 diabetes compared with the obese NGT group, after adjusting for race (given race-related differences in adiponectin) (20).

### Substrate utilization and metabolic flexibility across the glycemic spectrum.

RER was not significantly different among the 4 groups at baseline. Postabsorptive fat oxidation was not significantly different among the 4 groups before and after adjustment for race, sex, and Tanner stage (Table 2). After further adjustment for fat mass and lean mass, fat oxidation was lower in the NW compared with the other 3 groups (0.66 ± 0.09 mg/kg/min in NW vs. 0.91 ± 0.07 mg/kg/min in NGT, 0.86 ± 0.06 mg/kg/min in the prediabetes, and 0.97 ± 0.06 mg/kg/min in the type 2 diabetes groups, respectively, *P* = 0.004). Postabsorptive glucose oxidation was higher in the NW group compared with the other 3 groups. This difference persisted after further adjustment for fat mass and lean mass (*P* = 0.04).

Despite higher plasma fasting insulin concentrations, the rate of lipolysis was not significantly lower in the groups with obesity compared with the NW group; thus, adipose tissue insulin sensitivity was significantly lower in the groups with obesity across the spectrum of glycemia compared with the NW group (Table 2). Fasting FFA concentrations were significantly higher in the obese groups with dysglycemia compared with the other 2 groups (NW and obese NGT) (*P* < 0.001) (Table 2) and inversely related to adipose tissue insulin sensitivity (*r* = –0.46, *P* < 0.001).

**Figure 1:**
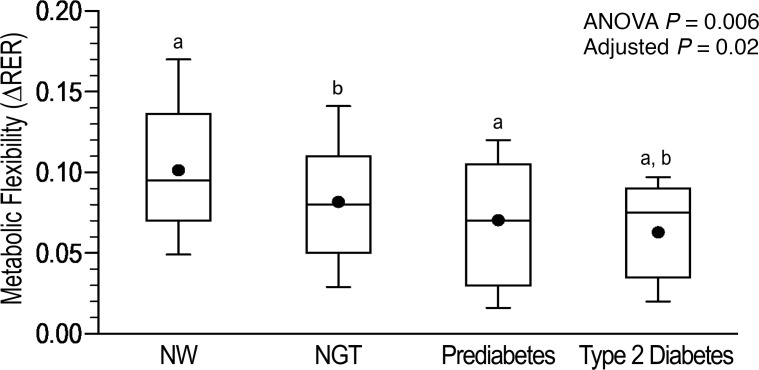
Metabolic flexibility (ΔRER) in youth with obesity across the spectrum of glycemic regulation [NGT (*n* = 24), prediabetes (*n* = 28), and type 2 diabetes (*n* = 28)] compared with NW (*n* = 24) peers. The box plots depict the minimum and maximum values (whiskers), the upper and lower quartiles, the median, and the mean (circle). The length of the box represents the interquartile range. One-way ANOVA. a, post hoc Bonferroni’s *P* < 0.05 in prediabetes vs. NW and in type 2 diabetes vs. NW; b, post hoc Bonferroni’s *P* < 0.05 in type 2 diabetes vs. NGT.

The groups with dysglycemia had less increase in glucose oxidation and less suppression in fat oxidation in response to hyperinsulinemia compared with the NGT and NW groups (Table 2). Metabolic flexibility (ΔRER) decreased from NW to obese with NGT, prediabetes, and type 2 diabetes groups (0.10 ± 0.04, 0.08 ± 0.04, 0.07 ± 0.04, and 0.06 ± 0.04 respectively, *P* = 0.006). ΔRER remained significantly lower in the type 2 diabetes and prediabetes groups compared with NW and in the type 2 diabetes compared with the NGT group, after adjusting for race, sex, and Tanner stage. ΔRER was not significantly different between the NW and obese NGT groups (Figure 1).

Under insulin-stimulated conditions, the oxidative and nonoxidative glucose disposal rates were significantly lower in the obese groups compared with NW and lower in the dysglycemia groups compared with obese NGT. Peripheral insulin sensitivity (total and adjusted per FFM) decreased across the spectrum of glycemia in the groups with obesity and was significantly lower than that of the NW group (Table 2). FFA concentrations continued to be significantly higher in the group with type 2 diabetes compared with the other 3 groups under the insulin-stimulated conditions of the HEC (Table 2).

### Relationship of metabolic flexibility to abdominal adiposity, HbA1c, adiponectin, and lipids.

ΔRER was not related to total body fat, fat mass, or lean mass but was inversely related to waist circumference (*r* = –0.23, *P* = 0.048) and to visceral adipose tissue (VAT) (*r* = –0.27, *P* = 0.02). The relationship to VAT was not significant (*r* = –0.21, *P* = 0.07) after adjusting for race. ΔRER was inversely related to HbA1c (*r* = –0.2, *P* = 0.049), triglycerides (*r* = –0.28, *P* = 0.04), and triglyceride/HDL-cholesterol ratio (*r* = –0.3, *P* = 0.002) and positively related to HDL-cholesterol (*r* = 0.2, *P* = 0.04) and adiponectin (*r* = 0.37, *P* < 0.001).

### Relationship of metabolic flexibility to glucose and lipid metabolism.

After adjusting for race, sex, and Tanner stage, fasting FFA concentration was inversely related to baseline and clamp steady-state glucose oxidation (*r* = –0.36, *P* < 0.001 for both). Fasting FFA was inversely related to fasting RER (*r* = –0.31, *P* = 0.002). Clamp FFA concentration was inversely related to insulin-stimulated glucose oxidation (*r* = –0.28, *P* = 0.005) and positively to fat oxidation (*r* = 0.21, *P* = 0.04).

After adjusting for race, sex, and Tanner stage, ΔRER was positively related to clamp steady-state glucose oxidation (*r* = 0.49, *P* < 0.001) and nonoxidative glucose disposal (*r* = 0.2, *P* = 0.06) and inversely related to fat oxidation (*r* = –0.44, *P* < 0.001). ΔRER also correlated positively with percentage suppression of FFA in response to hyperinsulinemia (*r* = 0.3, *P* = 0.01).

**Figure 2:**
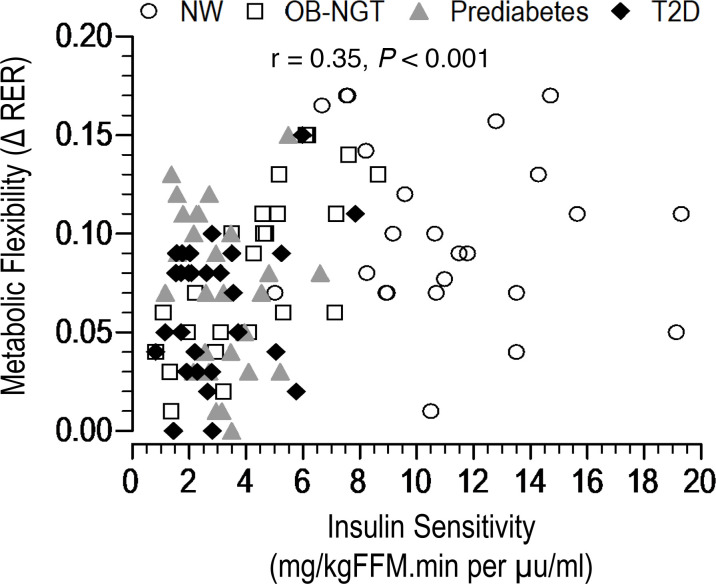
Relationship of insulin sensitivity to metabolic flexibility (ΔRER) in youth with NW (*n* = 24) and with obesity across the spectrum of glycemic regulation [NGT (*n* = 24), prediabetes (*n* = 28), and type 2 diabetes (*n* = 28)].

ΔRER correlated positively with insulin-stimulated glucose disposal (Rd_FFM_) (*r* = 0.35, *P* < 0.001) and with insulin sensitivity per FFM before (*r* = 0.35, *P* < 0.001) and after (*r* = 0.29, *P* = 0.003) adjusting for race, sex, and Tanner stage (Figure 2). The relationship of ΔRER to adipose tissue insulin sensitivity (IS) was not significant (*r* = 0.2, *P* = 0.08).

To identify significant independent determinants of metabolic flexibility, we performed multiple regression analysis. In a model with ΔRER as the dependent variable, IS_FFM_ (β = 0.4, *P* = 0.004) (or Rd_FFM_ [β = 0.30, *P* = 0.004]), percentage suppression of FFA (*r* = 0.26, *P* = 0.007), and race/ethnicity (β = –0.23, *P* = 0.02) contributed to the variance in ΔRER (*R*^2^ = 0.30, *P* < 0.001) independent of percentage body fat (or VAT), sex, Tanner stage, and HbA1c as covariates in the model.

## Discussion

We evaluated metabolic flexibility in youth with impaired glucose regulation (prediabetes and type 2 diabetes) compared with obese youth with normoglycemia and NW peers and evaluated the metabolic determinants of metabolic flexibility in these youth. Our study demonstrates that youth with prediabetes and type 2 diabetes have impaired metabolic flexibility compared with youth with obesity and normoglycemia and with NW. This metabolic inflexibility is related to impairment in glucose and lipid metabolism, with inflexibility in suppressing fat oxidation by insulin and a defect in glucose transport in youth with impaired glucose regulation. This suggests that metabolic inflexibility or defect in substrate utilization becomes manifest at the extreme of the metabolic phenotype in obese youth with more severe impairment in IS.

Our finding of no significant differences in metabolic flexibility between youth with NW and with obesity and NGT is consistent with the observation that youth with NW or obesity are able to adapt to changes in macronutrient intake and increase substrate oxidation (glucose vs. fat oxidation) in response to short-term changes in metabolic fuels through dietary manipulation (12, 13). However, our youth with prediabetes and type 2 diabetes manifested impairment in metabolic flexibility, with lower oxidative and nonoxidative glucose disposal and less suppression of fat oxidation in response to hyperinsulinemia, compared with the NGT and NW groups. Peripheral IS independently contributed to the variance in metabolic flexibility in our study. The marked reduction in glucose disposal and peripheral IS in youth with prediabetes and type 2 diabetes compared with similarly obese youth with NGT and with NW peers is consistent with our previous reports (15). In a study of obese youth with NGT pair matched for adiposity, those who were more insulin sensitive (above the median glucose disposal rate during the clamp) had greater metabolic flexibility (21). However, in the latter study, the reduced suppression of lipid oxidation during hyperinsulinemia did not differ in the insulin-sensitive versus -resistant groups (21). Perseghin et al. reported lower fat oxidation in the fasting state and less suppression in fat oxidation in response to glucose challenge in youth with nonalcoholic fatty liver disease compared with obese peers without fatty liver, indicating impaired metabolic flexibility in nonalcoholic fatty liver disease (22), a condition associated with reduced multiorgan IS (23, 24). In a previous study, we found that normoglycemic obese youth with severe versus moderate insulin resistance had less suppression of fat oxidation and less increase in glucose oxidation during the clamp (16). Our study advances these findings to youth across the glycemic spectrum and demonstrates that the defect in metabolic flexibility is associated with the degree of impairment of glucose disposal (i.e., IS), which is more evident in youth with prediabetes and type 2 diabetes.

Our findings are consistent with the findings of Galgani et al. in adults with type 2 diabetes compared with obese controls (5). In that study, the difference in metabolic flexibility between the 2 groups was no longer significant after adjusting for glucose disposal rate and was corrected after weight loss (5). The authors concluded that a defect in glucose transport was responsible for the metabolic inflexibility, rather than a primary defect in glucose oxidation. Similarly, in adults with type 2 diabetes compared with obese controls, insulin-stimulated glucose oxidation during the hyperinsulinemic clamp was determined by insulin-stimulated glucose uptake (25), suggesting that the primary defect is in substrate availability secondary to limitation in substrate transport. In vivo mitochondrial function (measured by phosphocreatine recovery kinetics) was related only to basal substrate oxidation and was not a significant predictor of insulin-stimulated metabolic flexibility (25). Nevertheless, skeletal muscle mitochondrial content was found to be a marker of metabolic flexibility (26).

Although HbA1c was related to ΔRER, the effect of glycemia was not significant in the multivariable analysis, suggesting that IS is the primary determinant of metabolic inflexibility in these youth early in the diabetes disease process and in relatively adequate glycemic control. These findings are consistent with those in adults where ΔRER correlated with IS after adjusting for glycemia but not after adjustment for FFA (27).

Another important finding is that fatty acid metabolism plays an important role in metabolic flexibility. The youth with obesity had significantly lower adipose tissue IS and higher fasting FFA compared with the NW group. Postabsorptive fat oxidation adjusted for lean and fat mass, Tanner stage, sex, and race was significantly higher in the youth with obesity compared with NW. This is consistent with prior reports of higher fat oxidation in children with obesity compared with NW and the importance of both lean and fat mass as determinants of fat oxidation (28). Higher fat oxidation in the obese state has been hypothesized to be a mechanism to limit further weight gain based on longitudinal studies in Pima Indians (29). This may still be operational in youth as opposed to adults with obesity in whom fasting lipid oxidation is lower than in lean individuals at the total body level (3) and across the leg muscle bed (3). Importantly, there was impairment of suppression of fat oxidation under hyperinsulinemic conditions and higher steady-state FFAs in the groups with dysglycemia compared with the groups with obesity and NGT and with NW. Similarly, lipolysis was found to be sensitive to insulin’s effect in obese and NW adolescents and adults, whereas basal FFAs and their suppression in response to the increment in insulin was reduced in adolescents with obesity compared with lean adults (30). In our study, the reduced suppression of FFAs under hyperinsulinemic conditions was independently related to lower ΔRER, supporting the contribution of impaired suppression of fat oxidation to the metabolic inflexibility in the youth with prediabetes and type 2 diabetes. An impaired capacity to regulate fat oxidation in response to high-fat feeding was reported in the obese insulin-resistant state (31). High-fat diet and increased fat flux in insulin-sensitive humans and in mice were associated with reduction in the expression of genes involved in oxidative phosphorylation, possibly through reduction in insulin signaling (32). Moreover, acute lipid infusion increasing FFAs is known to impair IS in adults and children (33, 34). Our results indicating an effect of FFA independent of IS on ΔRER are consistent with previous studies where elevated FFAs in adults with type 2 diabetes versus controls contributed to metabolic inflexibility (5, 25).

Our findings of impaired metabolic flexibility only in the youth with more extreme phenotype of impaired IS (prediabetes and type 2 diabetes) compared with NW, with no significant difference in metabolic flexibility between the groups with NW and with obesity with NGT, supports the conclusion that the primary defect in metabolic flexibility lies in substrate availability related to reduction in glucose uptake and impaired fatty acid metabolism (5, 25).

It remains unclear if metabolic inflexibility may be a primary cause of insulin resistance at least in some individuals with genetic predisposition. In support of this, studies in Pima Indians showed that family membership accounted for 28% of the variance in 24-hour room calorimetry respiratory quotient (RQ) and a high RQ (reflecting low ratio of fat to carbohydrate oxidation) was associated with subsequent weight gain (35). Also in Pima Indians, increased clamp lipid oxidation predicted diabetes prospectively, after adjustment for relevant confounders including glucose disposal, acute insulin response, age, sex, and body fat (36).

The limitations of this study are inherent to the cross-sectional study design, which limits the assessment of the evolution of the metabolic abnormalities. We used higher insulin infusion rate during the hyperinsulinemic clamp in the groups with obesity compared with the NW group. Despite the higher clamp insulin concentration in the groups with obesity, they had less responsiveness to insulin in substrate utilization, further supporting our conclusions. We accounted for the imbalance in racial/ethnic distribution of the study population in our statistical analyses. We found that metabolic flexibility appeared to be affected by race/ethnicity (lower in Hispanics). However, the race/ethnicity difference detected in this study will need to be verified in a more balanced population to evaluate racial differences in metabolism as a primary outcome. In adults, studies have found racial differences in metabolic flexibility, with AAs having higher ΔRER compared with Whites, after adjusting for IS and diabetes status (37).

In conclusion, metabolic inflexibility is a feature of a more severe metabolic phenotype in obese youth who have more severe impairment in IS and altered glucose metabolism, i.e., youth with prediabetes and type 2 diabetes, compared with youth with NW and with obesity and normoglycemia. This is related to a defect in substrate utilization associated with reduced skeletal muscle and adipose tissue IS. Our findings support the use of metabolic flexibility as an outcome measure in assessing metabolic risk and the response to interventions aiming at improving metabolism. Additional studies are needed to assess the predictive value of metabolic flexibility in metabolic risk.

## Methods

### Study design

#### Study participants.

A total of 104 adolescents, 15.6 ± 1.8 years of age, Tanner stages II–V, 29% NH Black, 12% NH White, and 59% Hispanic, participated in the study. They included 24 NW (BMI < 85% for age and sex) with NGT and 80 overweight (85th percentile ≤ BMI < 95th percentile) or obese (BMI ≥ 95th percentile) participants. Based on the oral glucose tolerance test (OGTT) results, study participants with overweight/obesity were categorized according to their glycemic status as having NGT (*n* = 24); prediabetes (*n* = 28) including impaired fasting glycemia (IFG), impaired glucose tolerance (IGT), or both (IGT/IFG); or type 2 diabetes (*n* = 28), based on the diagnostic criteria of the American Diabetes Association (38). Participants with type 2 diabetes were in adequate glycemic control (HbA1c < 8.5%). The mean duration of diabetes was 15.5 ± 16.8 months, median 8.95 months (range 0–71.2 months), treated with lifestyle changes (*n* = 6), metformin alone (*n* = 16), metformin and insulin (*n* = 4), or insulin alone (*n* = 2). Oral hypoglycemic agents and long-acting insulin therapy were discontinued 48 hours prior to study as before (15). Short-acting insulin was administered as necessary up to 6 hours prior to OGTT or clamp to maintain glycemic control. Participants were weight stable, not enrolled in scheduled physical activity or dietary intervention. They were instructed to refrain from participation in physical activity for 48 hours prior to admission to the research unit. They were excluded in the presence of other diseases or chronic medication that could interfere with endocrine function or if pregnant.

Metabolic experiments were performed in the Metabolic Research Unit (MRU) at the Children’s Nutrition Research Center. Participants were admitted to the MRU 24 hours prior to the clamp study. While the subjects were in the MRU, they consumed a standardized weight maintenance diet (55% carbohydrate, 30% protein, and 15% fat) prepared by the metabolic kitchen.

#### Anthropometric measurements.

Standard anthropometric measurements were measured from the eligible participants. Height was measured on a fixed wall stadiometer (Holtin Ltd.) to the nearest centimeter 3 times and then averaged. Weight was measured in light clothing to the nearest 0.1 kg on a balance scale. Waist circumference was measured at the midline from the inferior margin of the last rib and the crest of the ileum to the nearest 0.1 cm 3 times and averaged.

#### Body composition and abdominal fat measurement.

Body composition percentage body fat, fat mass, and FFM was determined by dual-energy x-ray absorptiometry scan. Abdominal fat distribution was obtained by MRI scan at L4–L5 in a subset (related to technical difficulties in scan acquisition).

#### OGTT.

Participants ingested a solution containing 1.75 g/kg body weight, max 75.0 g of dextrose. Blood samples were obtained at –15 minutes, at 0 minutes before, and at 15, 30, 60, 90, and 120 minutes after the ingestion, to determine plasma glucose and insulin. Fasting lipid profile, HbA1c level, and adiponectin levels were obtained.

#### HEC study.

Continuous indirect calorimetry was performed using the ventilated hood system (Sensormedics metabolic cart). Carbon dioxide production and oxygen consumption were measured after 12-hour fast at basal period (–30 to 0 minutes) in resting awake state and during the last 30 minutes of the HEC study (150–180 minutes). Urine was collected for measurement of urine nitrogen excretion (fasting and at the end of the clamp). We eliminated the first 5 minutes of the measurement and calculated the RER and substrate oxidation using formulas of Frayn, as described below.

For basal substrate turnover, basal total body lipolysis was evaluated by the use of stable isotope [^2^H_5_] glycerol started 120 minutes before starting the clamp experiment (15, 23). Arterialized blood samples for glucose, insulin, and isotopic enrichment were obtained before the start of the isotope infusion and every 15 minutes from 60 to 120 minutes. Turnover calculations were made over the last 30 minutes of the isotopic steady state to determine rate of lipolysis and adipose tissue IS (see calculations paragraph below).

Following the baseline isotopic infusion period, an HEC was performed (15) to evaluate in vivo insulin action. Intravenous insulin (Humulin R; Eli Lilly & Co.) was infused at a constant rate of 40 mU/m^2^/min for participants with a BMI < 85th percentile and 80 mU/m^2^/min for participants with a BMI ≥ 85th percentile, as before (39). An infusion of 20% dextrose was used to maintain plasma glucose clamped at approximately 100 mg/dL. The rate of glucose infusion was determined based on arterialized plasma glucose measurements every 5 minutes. Blood was sampled every 10–15 minutes for determination of insulin levels (15, 23, 39).

Regarding calculations, substrate turnover at baseline was calculated during the last 30 minutes of the fasting 2-hour isotopic infusion period according to steady-state tracer dilution equations to determine basal rate of lipolysis (23). Insulin-stimulated glucose disposal (Rd) was calculated during the last 30 minutes of the HEC and expressed per FFM (mg/min/kg_FFM_). Peripheral skeletal muscle IS was calculated by dividing the Rd by the steady-state clamp insulin concentration and expressed per FFM (mg/min/kg_FFM_ per μU/mL). Adipose tissue IS was calculated as the inverse of the product of glycerol rate of appearance in plasma and fasting plasma insulin concentration (16, 23, 40).

For substrate utilization and metabolic flexibility determination, glucose and fat oxidation were calculated according to Frayn’s equations (41). Nonoxidative glucose disposal (nonoxidative Rd) was calculated by subtracting glucose oxidation from the rate of glucose turnover. An increase in RER from baseline in response to insulin (ΔRER) was used as a measure of metabolic flexibility.

### Analytic methods

#### Isotope enrichment.

The tripropionate derivative of glycerol was prepared and isotopic enrichment of [^2^H_5_] glycerol measured by gas chromatography–mass spectrometry (Agilent Technologies 6890N GC, 5975 inert XL EI/CI MS) (23).

#### Biochemical measurements.

Plasma glucose was measured with a glucose analyzer (Yellow Springs Instrument, Thermo Fisher Scientific). Insulin levels were measured by electrochemiluminescence immunoassays (Elecsys 2010, Roche Diagnostics). HbA1c was measured using Tina-quant HbA1c immunoassay from Roche, and lipids were measured using the standards of the Centers for Disease Control and Prevention at LabCorp Inc. Adiponectin concentration was measured using MAGPIX (MILLIPLEX MAP) immunoassay (MilliporeSigma).

### Statistics

Descriptive statistics and distributional parameters were examined for all variables. Statistical analyses were performed using 1-way ANOVA followed by post hoc Bonferroni’s correction for multiple-group comparison. A Kruskal-Wallis test was used for multiple-group comparison of nonparametric variables. Categorical variables were compared using χ^2^ test. ANCOVA was used to adjust for group differences in race/ethnicity and important covariates (sex and Tanner stage). Bivariate relationships between metabolic flexibility (ΔRER) and physical and metabolic outcome variables were examined using Pearson’s or Spearman’s correlation and multivariable relationships using multiple regression analyses. Nonparametric variables were log transformed before the regression analyses. All analyses were performed using SPSS version 24 (SPSS Inc.). Data are presented as mean ± SD or as percentage as appropriate. *P* values with post hoc Bonferroni’s correction for multiple comparisons are presented. A 2-tailed *P* ≤ 0.05 was considered statistically significant.

### Study approval

The study received approval from the Institutional Review Board of Baylor College of Medicine. Parental informed consent and child assent were obtained in writing prior to any research procedure.

## Author contributions

FB designed and performed the study, obtained funding, analyzed the data, and wrote the manuscript. SKB and MP contributed to data collection and reviewed the manuscript. AA and SS contributed to laboratory data and reviewed the manuscript. All authors approved the manuscript in its final version. FB is the guarantor of this work and, as such, had full access to all the data in the study and takes responsibility for the integrity of the data and the accuracy of the data analysis.

## Supplementary Material

Trial reporting checklists

ICMJE disclosure forms

## Figures and Tables

**Table 1:**
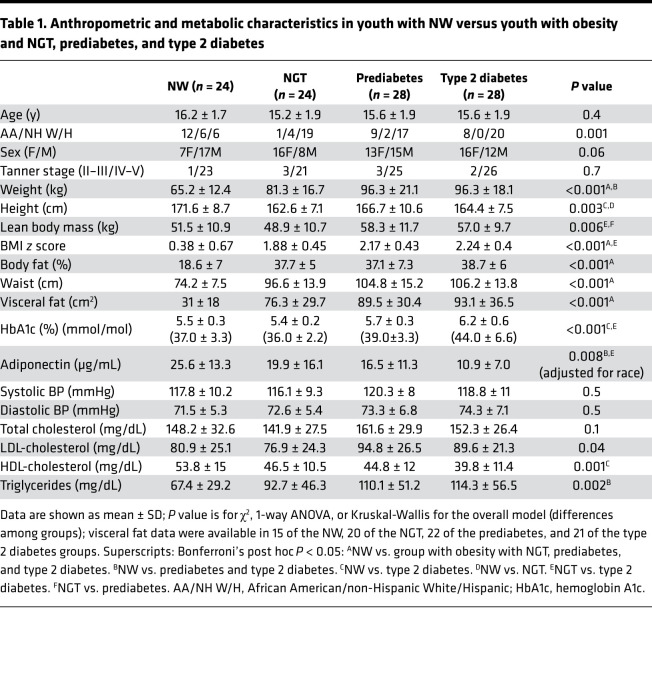
Anthropometric and metabolic characteristics in youth with NW versus youth with obesity and NGT, prediabetes, and type 2 diabetes

**Table 2:**
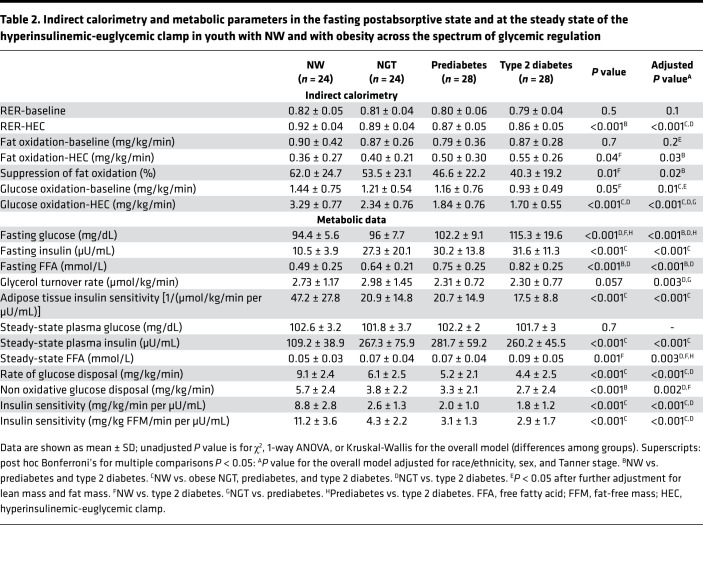
Indirect calorimetry and metabolic parameters in the fasting postabsorptive state and at the steady state of the hyperinsulinemic-euglycemic clamp in youth with NW and with obesity across the spectrum of glycemic regulation

## Version 1 02/22/2021

Electronic publication
