# Supplementary material for: Metabolic flexibility across the spectrum of glycemic regulation in youth
Source: JCI Insight. 2021 Feb 22;6(4):e146000. doi: 10.1172/jci.insight.146000 (PMC7934924; doi:10.1172/jci.insight.146000)
Supplement: Trial reporting checklists [file jciinsight-6-146000-s163.pdf]

STROBE Statement

	Item No	
**Title and abstract**	1	Provided in the abstract an informative and balanced summary of what was done and what was found
**Introduction**		
Background/rationale	2	Explained the scientific background and rationale for the investigation being reported
Objectives	3	Stated specific objectives and prespecified hypotheses
**Methods**		
Study design	4	Presented key elements of study design early in the paper
Setting	5	Described the setting, locations and data collection
Participants	6	Described the eligibility criteria, and the methods of selection of participants
Variables	7	Clearly defined all outcomes, predictors, potential confounders.
Data sources/ measurement	8	For each variable of interest, provided details of methods of assessment (measurement).
Bias	9	N/A
Study size	10	Statistical section and description of participants
Quantitative variables	11	Explained how quantitative variables were handled in the analyses. Described which groupings were chosen and why
Statistical methods	12	(*a*) Described all statistical methods
		(*b*) Described any methods used to examine subgroups and interactions
**Results**		
Participants	13	(a) Reported numbers of individuals recruited in each group and eligibility/exclusion criteria.
Descriptive data	14	(a) Gave characteristics of study participants and information on potential confounders
		(b) Indicated number of participants with missing data for each variable of interest
Outcome data	15	Described
Main results	16	(*a*) Gave unadjusted estimates and, if applicable, confounder-adjusted estimates and their precision (eg, 95% confidence interval). Make clear which confounders were adjusted for and why they were included
		(*b*) Reported category boundaries when continuous variables were categorized
Other analyses	17	Reported other analyses done—eg analyses of subgroups and interactions.
**Discussion**		
Key results	18	Summarised key results with reference to study objectives
Limitations	19	Discussed limitations of the study, taking into account sources of potential bias or imprecision.
Interpretation	20	Gave a cautious overall interpretation of results considering objectives, limitations, multiplicity of analyses, results from similar studies, and other relevant evidence
Generalisability	21	Discussed the generalisability (external validity) of the study results
**Other information**		
Funding	22	Gave the source of funding and the role of the funders for the present study and, if applicable, for the original study on which the present article is based
